# Efficacy and safety of citalopram versus amitriptyline in the treatment of major depression

**DOI:** 10.4103/0019-5545.55952

**Published:** 2005

**Authors:** Anand Mathur, D.K. Sharma, Ashok Choudhary, Mahendra Jain

**Affiliations:** * Professor and Head, Department of Psychiatry, J.L.N. Medical College and Deaddiction Centre, Ajmer 205001; ** Associate Professor, Department of Psychiatry, J.L.N. Medical College and Deaddiction Centre, Ajmer 205001; *** Assistant Professor, Department of Psychiatry, J.L.N. Medical College and Deaddiction Centre, Ajmer 205001; **** Assistant Professor, Department of Psychiatry, J.L.N. Medical College and Deaddiction Centre, Ajmer 205001

**Keywords:** Major depression, side-effects, tricyclic antidepressants, SSRIs

## Abstract

**Background:**

Double-blind clinical trials comparing citalopram with amitriptyline or other tricyclic antidepressants are lacking in India.

**Aim:**

To evaluate the efficacy and safety of the newer antidepressant citalopram in the treatment of major depression.

**Methods:**

The clinical acceptability and safety profile of citalopram was assessed and compared with that of amitriptyline in 40 patients in an outpatient set-up. Patients aged 18 to 65 years who fulfilled the diagnostic criteria for a single or recurrent major depressive disorder (as defined by DSM-IV) for a minimum of 2 weeks were enrolled. Patient assessment was done at screening, baseline, end of week 1, week 2, week 3, week 4, week 5 and week 6 for efficacy and safety parameters such as Hamilton Depression Rating Scale (HDRS), Clinical Global Impression (CGI) Scale, adverse event follow up, blood pressure and pulse. Three-level statistical analysis including ANOVA was performed on all efficacy measures.

**Results:**

On the HDRS the percentage reduction in the mean score for the citalopram group (Group 1) was 72.12%, while that for the amitriptyline group (Group 2) was 67.93%. On the CGI-Improvement Scale, the percentage reduction at the end of the study for the citalopram group was 56.79% while in the amitriptyline group it was 44.70%. Twenty per cent of patients in Group 1 reported adverse events compared to 75% in Group 2.

**Conclusions:**

Citalopram is effective in the treatment of major depression at the dosages range of 20–60 mg/day and its efficacy is equivalent to that of standard tricyclic antidepressants such as amitriptyline, with a substantially better tolerability profile.

## INTRODUCTION

Depression is a chronic recurrent disorder and requires long-term treatment with drugs that have a low incidence of adverse effects. Traditional tricyclic antidepressants (TCAs) are now being replaced by the new selective serotonin reuptake inhibitors (SSRIs), which have minimal psychomotor and anticholinergic effects, and fewer problems such as weight gain and cardiotoxicity.

Citalopram—a ‘second-generation’ antidepressant drug like fluvoxamine, fluoxetine and paroxetine—enhances serotoninergic neurotransmission through highly selective and potent inhibition of neuronal serotonin reuptake.^1^ Its major metabolites possess similar pharmacological properties but are weaker and selective inhibition of serotonin uptake is less. *In vitro*, citalopram blocks serotonin uptake in platelets, synaptosomal preparations and brain slices. It competes with serotonin and imipramine for a common binding site.

The antidepressant efficacy of citalopram has been well documented in clinical trials. Non-comparative studies over 4–6 weeks have shown that citalopram produces marked improvement in 30%–70% of patients and moderate improvement in another 10%–50% using the Hamilton Depression Rating Scale (HSRS) and Clinical Global Impression (CGI) Scale.^2^

A number of open clinical studies have shown citalopram to be an effective antidepressant with fewer undesirable side-effects.^3^–^5^ Unlike TCAs, citalopram is devoid of anti-adrenegic, antihistaminic and anticholinergic properties. Mild and transient nausea with or without vomiting is the most frequent adverse effect of citalopram.

Double-blind comparative clinical trials indicate that citalopram is as effective as TCAs such as amitriptyline^6^–^9^ or imipramine.^10^,^11^ However, amitriptyline- or imipramine-treated patients reported a higher frequency of adverse events, especially anticholinergic side-effects than did the citalopram-treated patients.

We took up this study because such double-blind clinical trials comparing citalopram with amitriptyline or other TCAs are lacking in India.

## METHODS

A total of 40 patients aged 18–65 years, who fulfilled the diagnostic criteria for a single or recurrent major depressive disorder, as defined by DSM-IV for a minimum of 2 weeks, were enrolled at the Department of Psychitry, J.L.N. Medical College and Hospital, Ajmer. The nature of depressive disorders was moderate or severe, without mood-incongruent psychotic features. Pregnant or nursing women were not included in the study, and women of childbearing age were advised to use appropriate birth control methods during the trial period. All the patients signed an informed consent form before their inclusion in the study.

Only OPD patients with a minimum total score of 15 on the 17-item HDRS at both the initial screening and pretreatment baseline were included in the study. Patients with a history of alcohol dependence or substance abuse during the past 2 years, who showed a placebo response during screening (i.e. ≥20% decrease in HDRS score between screening and baseline) or those who showed acute or unstable medical problems were excluded from the study. The other exclusion criteria were: hypersensitivity to SSRIs previous use of citalopram, history of seizures, concomitant psychotropic medication, bipolar depression, other significant organic disease, clinically significant laboratory abnor-malities, or other primary psychiatric diagnoses.

### Study design

Patients meeting the initial inclusion criteria entered a one-week placebo screening phase. At the end of the one-week placebo washout period, patients returned for the final screening procedure. All the patients who satisfied the final inclusion and exclusion criteria were then randomized to either citalopram (Group 1) or amitriptyline (Group 2) treatment in an open, parallel-group study design.

The study drug was given in bottles containing 7 days' supply. Patients in Group 1 received citalopram 20 mg tablets, and patients in Group 2 received amitriptyline 25 mg tablets. The initial dose of citalopram in Group 1 was 20 mg daily for the first 2 weeks, which, based on response and tolerance, was increased to 60 mg daily. The initial dose of amitriptyline in Group 2 was 25 mg tid (75 mg/day) which, based on response and tolerance, was increased to 150 mg/day.

Laboratory investigations (urine analysis, haematology and biochemistry) were conducted by an approved professional laboratory at screening and at the end of the study.

During the study, the trial drug was kept in a secure place and not supplied to anyone expect the co-investigators or deputy involved in the study. A detailed account of use of the drug with the date and patient number was maintained. Unused drug was returned to the monitor after the study was completed.

Full information concerning the name, dosage and duration of other concomitant therapy was recorded.

### Adverse event management

At each follow-up visit, the patients were asked about likely adverse events. Any adverse events reported were recorded in the adverse event form. Any serious/life-threatening side-effects were to be informed to the sponsor's representative immediately. Details of adverse event management (corrective therapy, change in dosage, withdrawal of drug, etc.) were clearly recorded, including the severity of side-effects.

The number and percentage of patients experiencing each specific event for treatment-emergent signs and symptoms (TESS) (defined as experience that appeared for the first time during the study) were calculated for both the groups.

### Patient assessment

Patients were assessed at screening, baseline, end of week 1, week 2, week 3, week 4, week 5 and week 6 for the efficacy and safety parameters—HDRS, CGI, adverse event follow-up, blood pressure and pulse.

A general physical examination was conducted, and medical and psychiatric history recorded only at screening. Clinical laboratory evaluations were made at screening and at the end of the trial.

The primary efficacy variables were the 17-item HDRS and the CGI-Improvement Scale. Responder status was defined as improvement during treatment of ≥50% on the HDRS total scores. In the case of the CGI-Improvement Scale, responder status was defined as improvement to a score of 1 (very much improved) or 2 (much improved). A sustained response was defined as improvement that, once observed, persisted until the end of trial. A final 21-item HDRS total score of 8 or less was defined as remission.

### Statistical methods

Three basic statistical analyses were performed on all efficacy measures. An analysis of variance (ANOVA) for baseline ratings was done to assess the equivalence of the treatment groups at the beginning of the study. Pre-treatment versus post-treatment ANOVA was done to examine the response produced by each drug over time. An ANOVA for each assessment was done to evaluate the differences between the treatment groups.

## RESULTS

### Patient disposition

Forty patients who fulfilled the inclusion criteria were recruited for the trial. Initially, the citalopram group (Group 1) consisted of 20 patients and the amitriptyline group (Group 2) of 20 patients. All randomized patients who received the study drug comprised the intent-to-treat safety population. There were no drop-outs in either group, resulting in an intent-to-treat efficacy population of 20 patients (100%) in Group 1 and 20 (100%) in Group 2.

### Demographic and baseline characteristics

Only physically healthy patients were enrolled for the study. No significant differences were detected between the citalopram and amitriptyline groups on any demographic, diagnostic or psychiatric history variables (Table 1).

**Table 1:** Baseline demographic data of the intent-to-treat patient sample

Variable	Group 1 (citalopram) (*n*=20)	Group 2 (amitriptyline) (*n*=20)
Sex		
Male	9	7
Female	11	13
Age (years)		
18–30	8	5
31–40	5	7
41–50	6	6
More than 50	1	2
Mean baseline		
HDRS	25.65±0.22	25.10±2.88
CGI	4.05±0.22	4.25±1.12

HDRS: Hamilton Depression Rating Scale; CGI: Clinical Global Impression Scale

The distribution across age ranges showed that the majority of patients (32.5%) were in the 18–30 years' age group. Mean baseline HDRS scores were comparable among both the groups (25.65 for Group 1 vs 25.10 for Group 2).

### Treatment

The average daily dosage range of citalopram at the end of the trial was 36.00±16.67 mg and for amitriptyline it was 150.00±0.00 mg (Table 2).

**Table 2:** Mean daily dose (mg/day)

	Week					
	
Treatment group	1	2	3	4	5	6
Citalopram (mg) (Group 1)	20.00±0.00	20.00±0.00	27.00±9.79	25.00±8.89	35.00±17.01	36.00±16.67
Amitriptyline (mg) (Group 2)	75.00±0.00	86.25±27.48	101.25±36.70	150.00±0.00	150.00±0.00	150.00±0.00

### Primary efficacy

#### Hamilton Depression Rating Scale (HDRS)

**Fig. 1:**
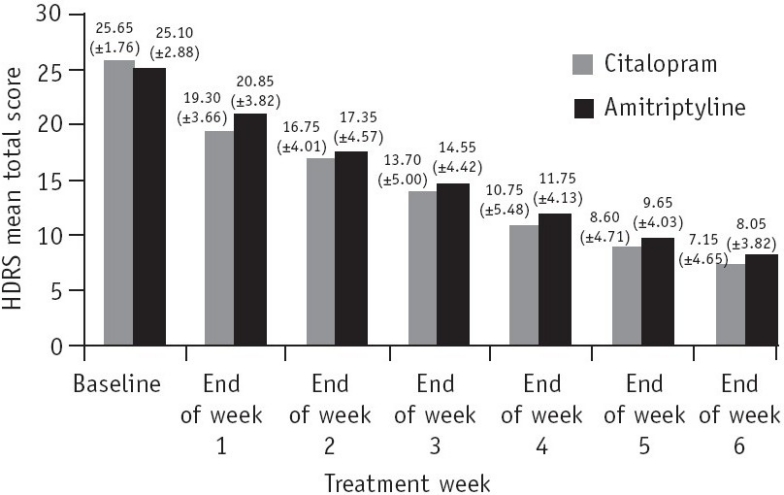
HDRS mean total score for Group 1 (citalopram) and Group 2 (amitriptyline) patients

Both the groups were comparable in levels of depression as measured by the HDRS total score at baseline. A summary of total score changes from baseline to end-point in HDRS is shown in Fig. 1.

By the end of week 3, Group 1 showed a mean decrease of more than 11.95 points on the HDRS total score, while Group 2 showed a mean decrease of 10.55. By the end of week 6 (end-point) the total reductions in the HDRS score as compared to baseline were 18.5 and 17.05 for the citalopram and amitriptyline groups, respectively. The percentage reduction in the mean HDRS score for the citalopram group was 72.12% while that for the amitriptyline group was 67.93%.

#### Clinical Global Impression (CGI)-Improvement Scale

**Fig. 2:**
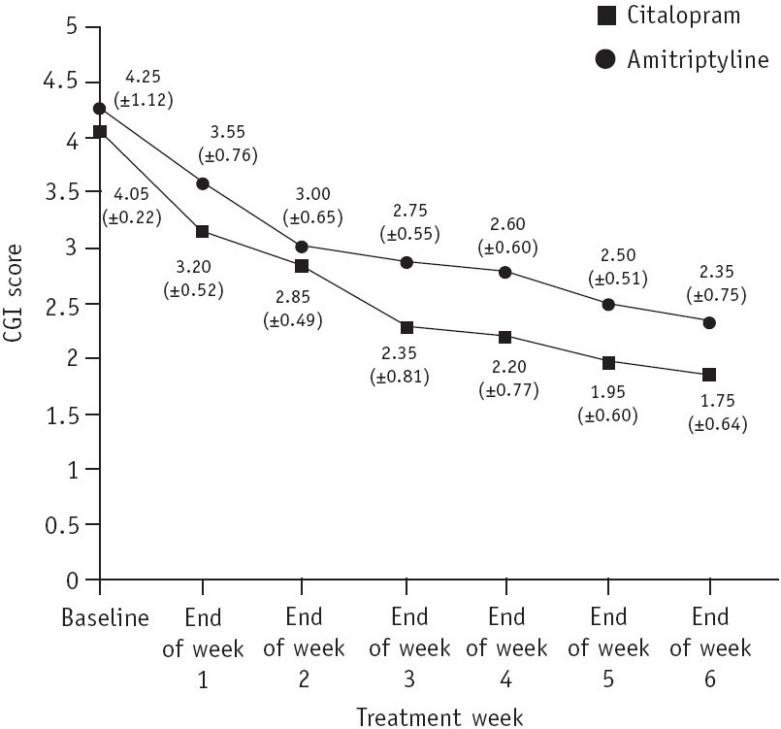
CGI mean scores for Group 1 (citalopram) and Group 2 (amitriptyline) patients

Both the groups were comparable in total CGI score at baseline. A summary of total score changes from baseline to end-point in the CGI Scale is shown in Figure 2. The percentage reduction in the mean CGI score for Group 1 (citalopram) was 56.79% while that for Group 2 (amitriptyline) was 44.70%.

### Tolerability

Treatment-emergent adverse events were reported by 20% (*n*=4) of Group 1 patients and 75% (*n*=15) of Group 2 patients.

The most common events are summarized in Table 3. The majority of adverse events in both treatment groups were mild to moderate and did not lead to discontinuation of treatment.

**Table 3:** Most frequently reported adverse events associated with citalopram and amitriptyline treatment (intent-to-treat safety sample)

	Number of patients reporting adverse events	
	
Adverse event	Group 1 (citalopram) *n*=20 (%)	Group 2 (amitriptyline) *n*=20 (%)
Nausea	2 (10)	0 (0)
Headache	1 (5)	0 (0)
Dry mouth	2 (10)	10 (50)
Erectile dysfunction	0 (0)	2 (10)
Drowsiness	0 (0)	7 (35)
Blurring of vision	0 (0)	1 (5)
Giddiness	0 (0)	3 (15)
Insomnia	0 (0)	3 (15)
Rashes	0 (0)	1 (5)
Anxiety	0 (0)	1 (5)

#### Overall assessment of tolerability

Overall assessment of tolerability was made at the end of the study by the investigator as well as the patient. This assessment was based on the number and severity of adverse effects and likelihood of a causal relationship—the drug could be assessed as having excellent, good, moderate or bad tolerability.

**Fig. 3:**
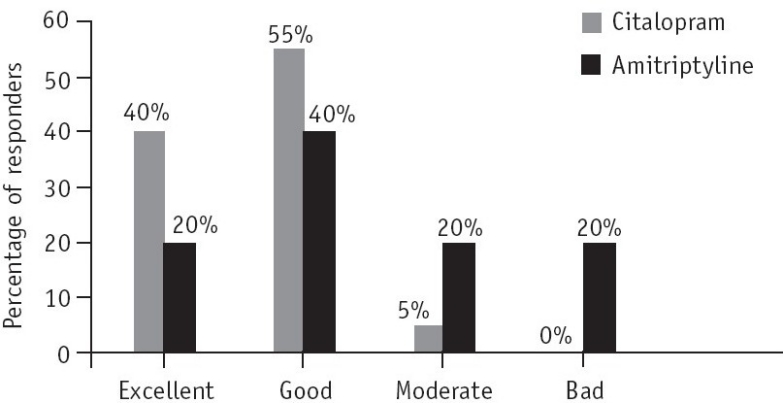
Overall assessment of tolerability by the investigator at the end of the 6-week study (assessments were based on the number and severity of side-effects and likelihood of a causal relationship).

In Group 1 (citalopram), according to the investigator's evaluation, 40% (*n*=8) of the patients showed excellent tolerability compared to 20% (*n*=4) of the patients in Group 2 (amitriptyline) (Fig. 3). No adverse event was reported by any patient in the citalopram group.

**Fig. 4:**
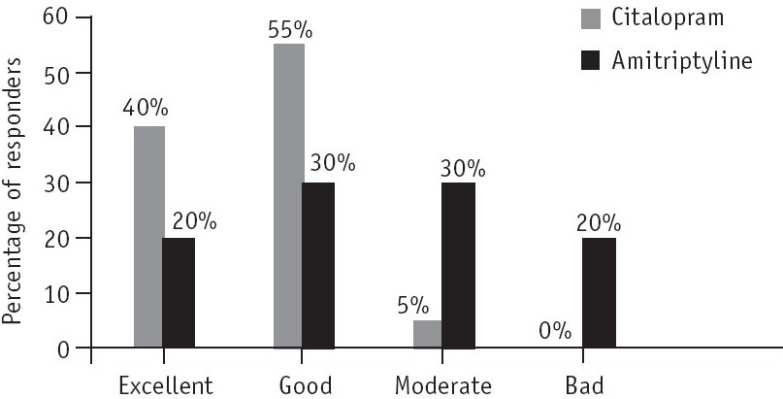
Overall assessment of tolerability by the patient at the end of the 6-week study (assessments were based on the number and severity of side-effects and likelihood of a causal relationship).

In the overall assessment of tolerability by the patient, 40% (*n*=8) in the citalopram group stated that the drug had excellent tolerability and 55% (*n*=11) stated that it had good tolerability (Fig. 4).

In the amitriptyline group 20% (*n*=4) of the patients reported excellent tolerability and 30% (*n*=6) reported good tolerability.

#### Overall evaluation of efficacy by the investigator

For the overall assessment of treatment acceptability and efficacy the protocol defined four categories, i.e. very good, good, moderate, unchanged or worse.

**Fig. 5:**
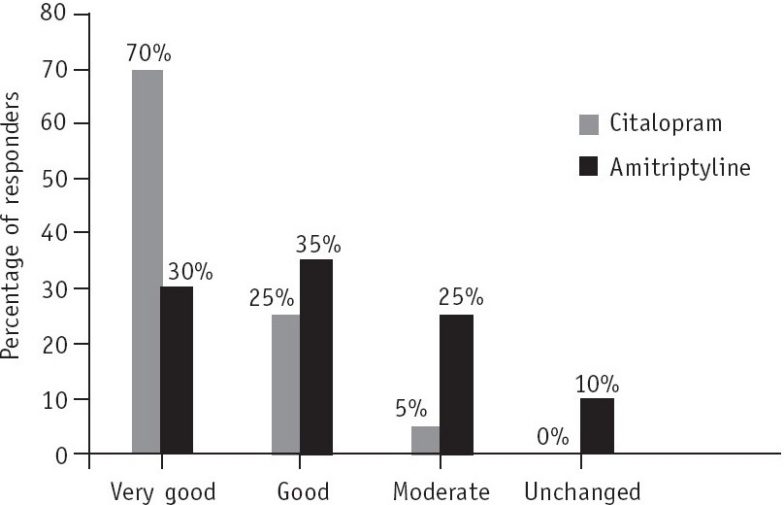
Overall assessment of treatment acceptability at the end of the 6-week study

In the citalopram group 70% (*n*=14) showed very good response, 25% (*n*=5) showed good response, 5% (*n*=1) showed moderate response. No patient in this group showed worse response (Fig. 5).

## DISCUSSION

The results of the study indicate that citalopram is effective in the treatment of depression at a dosage range of 20–60 mg/day.

In several double-blind clinical trials citalopram has demonstrated efficacy equivalent to that of the commonly prescribed TCA amitriptyline.^6^–^8^ A significant reduction in the HDRS score was observed in both the treatment groups at the end of 3 weeks proving that both the drugs are effective in reducing the symptoms of depression.

Though large differences in efficacy between the two treatment groups were not observed, the primary treatment outcome of this study suggests that citalopram is slightly more efficacious than amitriptyline as measured by the HDRS score throughout the 6-week period. In the secondary efficacy variable (CGI score) citalopram showed a slight benefit over amitriptyline.

In terms of the side-effect profile, citalopram was better tolerated than amitriptyline. More troublesome CNS and anticholinergic side-effects were observed with amitriptyline, which included dry mouth, erectile dysfunction, drowsiness, blurring of vision, giddiness, insomnia, anxiety and skin rashes. Fewer side-effects were observed with citalopram in a small number of patients, i.e. nausea in 2 (10%) and headache in 1 patient (5%). Our findings are in line with the pooled data from comparative clinical trials,^12^ which showed that citalopram is associated with an approximately 5% greater incidence of nausea and ejaculatory failure than TCAs. In contrast, dry mouth, increased sweating, tremor, somnolence, constipation, abnormalities of accommodation, dizziness, postural hypotension, palpitation and alteration in taste occurred significantly more frequently with TCAs and tetracyclics than with citalopram.

Thus, it can be concluded that the efficacy of citalopram is equivalent to that of amitriptyline, a standard TCA, in the treatment of outpatients with depression. Citalopram has a substantially better tolerability profile.
